# Genome size estimation from long read overlaps

**DOI:** 10.1093/bioinformatics/btaf593

**Published:** 2025-11-06

**Authors:** Michael B Hall, Chenxi Zhou, Lachlan J M Coin

**Affiliations:** Department of Microbiology and Immunology, The University of Melbourne, at the Peter Doherty Institute for Infection and Immunity, Melbourne, VIC, 3000, Australia; Department of Genetics, The University of Cambridge, Cambridge, CB2 3EH, United Kingdom; Department of Microbiology and Immunology, The University of Melbourne, at the Peter Doherty Institute for Infection and Immunity, Melbourne, VIC, 3000, Australia; Department of Clinical Pathology, The University of Melbourne, Parkville, VIC, 3010, Australia

## Abstract

**Motivation:**

Accurate genome size estimation is an important component of genomic analyses such as assembly and coverage calculation, though existing tools are primarily optimized for short-read data.

**Results:**

We present LRGE, a novel tool that uses read-to-read overlap information to estimate genome size in a reference-free manner. LRGE calculates per-read genome size estimates by analysing the expected number of overlaps for each read, considering read lengths and a minimum overlap threshold. The final size is taken as the median of these estimates, ensuring robustness to outliers such as reads with no overlaps. Additionally, LRGE provides an expected confidence range for the estimate. We validate LRGE on a large, diverse bacterial dataset and confirm it generalizes to eukaryotic datasets. On bacterial genomes, LRGE outperforms *k*-mer-based methods in both accuracy and computational efficiency and produces genome size estimates comparable to those from assembly-based approaches, like Raven, while using significantly less computational resources.

**Availability and implementation:**

Our method, LRGE (**L**ong **R**ead-based **G**enome size **E**stimation from overlaps), is implemented in Rust and is available as a precompiled binary for most architectures, a Bioconda package, a prebuilt container image, and a crates.io package as a binary (lrge) or library (liblrge). The source code is available at https://github.com/mbhall88/lrge and an archive at https://doi.org/10.5281/zenodo.17183812 under an MIT license.

## 1 Introduction

Genome size is a fundamental genomic characteristic essential for downstream analyses such as genome assembly (Wick and Holt 2020) and evolutionary studies (Pellicer and Leitch 2020). Accurate genome size estimation remains challenging, especially for non-model organisms and datasets with high heterogeneity or repetitive content. Existing methods primarily focus on short-read data and often require high computational resources or rely on pre-assembled references (Vurture *et al.* 2017, Ranallo-Benavidez *et al.* 2020), limiting their applicability to modern long-read sequencing platforms from Pacific Biosciences (PacBio) and Oxford Nanopore Technologies (ONT).

The advancement of long-read sequencing technologies has made it relatively easy to generate high-quality bacterial genome assemblies (Wick *et al.* 2023). Combined with the ever-increasing throughput of sequencing, automated pipelines for tasks like variant calling and genome assembly are now common (Petit and Read 2020, Murigneux *et al.* 2021, Bouras *et al.* 2024). These pipelines often require a genome size estimation from the user (Murigneux *et al.* 2021), or optionally estimate one (Petit and Read 2020, Bouras *et al.* 2024). However, existing size estimation tools are typically designed for short reads (Vurture *et al.* 2017, Ranallo-Benavidez *et al.* 2020) and struggle with the higher error rates of long reads, which can introduce many erroneous *k*-mers, the basis for some estimation methods (Ondov *et al.* 2016, Ranallo-Benavidez *et al.* 2020). Additionally, determining sequencing depth requires knowledge of the genome size (Hall 2022), so pipelines that downsample sequencing data to improve computational efficiency must either rely on user-provided size estimates or calculate them automatically.

Here, we present a novel approach leveraging long-read overlap data obtained from minimap2 (Li 2018) to provide accurate genome size estimates in a reference-free manner, without a reliance on *k*-mers. By focusing on read-to-read overlaps, our method efficiently captures genome-wide patterns of coverage and redundancy, offering a robust alternative to *k*-mer-based and assembly-dependent techniques. This tool is designed for analysing long-read datasets, making genome size estimation accessible for a broader range of organisms and experimental contexts.

Our method, LRGE, estimates genome size by analysing how individual reads overlap with one another. For each query read, it calculates the expected number of overlaps with a set of target reads, considering both the lengths of the reads and a minimum overlap threshold. Using this overlap information, the method derives a genome size estimate for each query read. These per-read estimates are then aggregated using the median, ensuring robustness to outliers such as reads with no overlaps, which would otherwise produce infinite estimates. This approach leverages the statistical properties of read overlaps to infer genome size without requiring a pre-assembled reference.

## 2 Methods

Suppose the genome size is **GS**. We assume that we have sequenced a set of |T| target reads *T* with average length ${\bar{\ell }}_{T}=\frac{1}{\left|T\right|}\sum_{t\in T}\ell (t)$, where ℓ(t) is the length of a specific read t∈T; as well as a second set of |Q| query reads *Q* with average read length ${\bar{\ell }}_{Q}$, defined in the same manner as ${\bar{\ell }}_{T}$. We denote by ov(Q,T) the set of reads in *Q* which overlap with *T* and by T∖{q} the set of reads in *T* not including *q*, which enables us to consider the case that *Q* and *T* may include the same reads. We make the assumption that reads in ov(Q,T) overlap due to originating from neighbouring regions in the genome and not due to repetitive DNA elements. To minimise the impact of repetitive DNA we provide an option to filter alignments due to internal matches (Li 2016), for use with eukaryotic genomes. We consider internal matches to be those overlaps where one read is fully contained within another.

If the target read *t* is located at a known position on the genome, how often would a randomly placed query read *q*, drawn from the GS−ℓ(q) possible positions, be expected to overlap with *t*? If we consider that the target read starts at genome position s(t) and ends at e(t) then to achieve an overlap of at least **OT** bases, the query read start position s(q) must lie within the interval


[s(t)+OT−ℓ(q),e(t)+1−OT].

The size of this interval is ℓ(q)+ℓ(t)−2*OT+1. We make an implicit assumption here that s(t)≥(ℓ(q)−OT) and that e(t)<GS−(ℓ(q)−OT), which holds for the vast majority of reads *t*. In the edge cases where this assumption does not hold, the size of the interval is reduced, with lower bound of ℓ(q)−OT, however we will ignore this edge effect from here onwards. The total number of available positions for the query read to start is GS−ℓ(q). Thus the probability that a read *t* overlaps with a distinct read *q* is equal to


(1) $$P(|\mathrm{ov}(\{t\},\{q\})|>0)=\frac{\ell (q)+\ell (t)-2\cdot \mathrm{OT}+1}{\mathrm{GS}-\ell (q)}$$

where we approximate **OT** as the base-pair threshold on the minimal overlap length, as defined by the minimap2 minimum chaining score, -m, (default 100 bp for overlaps) (Li 2018). The expected count of overlaps is thus given by


(2) $$E(|\mathrm{ov}(T\setminus \{q\},\{q\})|)=|T\setminus \{q\}|\frac{\ell (q)+{\bar{\ell }}_{T\setminus \{q\}}-2\cdot \mathrm{OT}+1}{\mathrm{GS}-\ell (q)}$$

which gives us a per-read estimate of **GS**,


(3) $$GS_{T,q}\approx \ell (q)+|T\setminus \{q\}|\frac{\ell (q)+{\bar{\ell }}_{T\setminus \{q\}}-2\cdot \mathrm{OT}+1}{\left|\mathrm{ov}(T\setminus \{q\},\{q\})\right|}$$

We calculate the overall genome size estimate, $GS_{T,Q}$, as the **median** of the defined per-read estimates:


(4) $$GS_{T,Q}=\text{median}\left(\left\{GS_{T,q}|q\in Q\wedge |\mathrm{ov}(T\setminus \{q\},\{q\})|>0\right\}\right)$$

as a read *q* with no overlaps will return an undefined per-read estimate.

### 2.1 Implementation

We have implemented this estimation process in the software tool LRGE using the Rust programming language, with overlaps being generated by minimap2 (Li 2018). LRGE offers a command-line application, along with a library API for use in other Rust projects.

The implementation provides two strategies for estimation: Two-set (*2set*), whereby *Q* and *T* are disjoint sets of reads; and all-vs-all (*ava*), where *Q* and *T* are identical sets of reads.

The *2set* strategy has the advantage where *Q* can be made smaller than *T* to reduce the number of per-read estimates, $GS_{T,q}$, as we ultimately take the median of these estimates. Additionally, it enables internally swapping *Q* and *T* so that the smaller dataset can serve as the reference for minimap2 alignment. While this does not affect the results, it significantly reduces the memory footprint during mapping when *T* is much larger than *Q*. This strategy is the default used in LRGE, with |Q|=5000 and |*T*|=10 000.

### 2.2 Evaluation

We compared the genome size estimations from LRGE using the *ava* and *2set* strategies, as well as GenomeScope2 (Ranallo-Benavidez *et al.* 2020), Mash (Ondov *et al.* 2016), and Raven (Vaser and Šikić 2021).

For LRGE *ava* we used 25 000 randomly selected reads, while for the *2set* strategy, we used a target set size of 10 000 and a query set size of 5000.

Mash (v2.3; Ondov *et al.* 2016) estimates were gathered using the subcommand sketch with the minimum *k*-mer copy number for filtering set to 10 and a sketch size of 100 000.

The parameters used for LRGE and Mash were selected based on a sweep of parameter combinations on a validation set described in the appendix (Parameter exploration).

To generate GenomeScope2 estimates, we computed the histogram of *k*-mer frequencies using KMC (v3.2; Kokot *et al.* 2017) with a *k*-mer size of 21, a minimum *k*-mer count (-ci) of 2, and a maximum *k*-mer count (-cs) of 10 000. These parameters were selected as they are the default recommended in the GenomeScope2 repository. We then ran GenomeScope2 (v2.0.1; Ranallo-Benavidez *et al.* 2020) against the frequency histogram with the same settings, with ploidy of 1.

Raven is a genome assembly tool that is designed to be time- and memory-efficient and uses an overlap-layout-consensus approach (Vaser and Šikić 2021)—keeping with the overlap theme. We set Raven (v1.8.3) to perform no polishing (-p 0) and use the size of the computed assembly as the ‘estimate’.

We evaluated the accuracy of each method using relative error, $\epsilon_{\text{rel}}$, which measures the percentage difference between the estimated value ($\hat{G}$) and the true value (*G*), scaled relative to the true value. It quantifies how close the estimate is to the true value, with positive values indicating overestimation and negative values indicating underestimation. It is defined as


(5) $$\epsilon_{\text{rel}}=\left(\frac{\hat{G}}{G}-1\right)\cdot 100$$

Statistical testing of the absolute relative error, CPU time, and maximum memory usage differences between methods were performed using a pairwise Tukey’s range test. For the absolute relative error testing, we only compared between the same sequencing technology—i.e. we do not compare PacBio for method A with ONT for method B.

#### 2.2.1 Prokaryote evaluation

We performed a large-scale evaluation of the estimation methods using a set of 3370 publicly available bacterial long-read sequencing runs—2468 ONT and 902 PacBio. These runs were selected as they were associated with a high-quality RefSeq assembly, which we took to be the true size. A detailed description of the data collection and filtering performed is provided in the appendix (Dataset selection). All experiments for prokaryotic samples were conducted on a server running Red Hat Enterprise Linux (RHEL) 9.4 equipped with Intel ^®^ Xeon ^®^ Gold 6448H CPUs.

#### 2.2.2 Eukaryote evaluation

While the primary development focus for LRGE was bacterial genomes, we investigated how the method generalizes to eukaryotes, such as multichromosome and diploid organisms. We used ONT reads from four model organisms with a high-quality RefSeq genome: *Saccharomyces cerevisiae*, *Drosophila melanogaster*, *Arabidopsis thaliana* and *Homo sapiens*.

Due to the larger genome sizes of these organisms, we used 10 000 query reads and 20 000 target reads for the *2set* strategy on *S. cerevisiae* and default (25 000) for the *ava* strategy. For *D. melanogaster* and *A. thaliana*, we used 100 000 target reads and 50 000 query reads for the LRGE *2set* approach and 100 000 reads for the *ava* strategy. For *H. sapiens*, we used 100 000 query and 2 000 000 target reads for the *2set* strategy and did not run *ava* strategy due to memory constraints. We enabled the -F option to filter out internal matches (overlaps where one read is fully contained within another) and—use-min-ref option to allow the smaller *Q* dataset to be used as reference for minimap2 alignment across all these eukaryotic datasets. The parameters were kept the same as for the prokaryote evaluation for all other methods. All experiments for eukaryote samples were conducted on a server running Ubuntu 22.04, equipped with a 2.90 GHz Intel ^®^ Xeon ^®^ Gold 6226R CPU, with the system recognizing up to 64 logical processors.

## 3 Results

To evaluate the accuracy of the estimations produced by LRGE, we collected publicly available bacterial long-read sequencing runs with associated high-quality reference assemblies (see Prokaryote evaluation). We benchmarked the two LRGE strategies against three other methods: Mash, which uses sketching of *k*-mers to produce an estimate; GenomeScope2, which performs statistical analyses on *k*-mer frequency spectra; and Raven, which is a rapid genome assembly method (see Evaluation).

Our primary metric of comparison is relative error ($\epsilon_{\text{rel}}$; Equation 5), which measures the percentage difference between the estimate and the true value, scaled by the true value. For example, a $\epsilon_{\text{rel}}$ of 50% for a sample with a genome size of 2 Mbp indicates the estimate is over by half the true size—3 Mbp. To simplify the evaluation, we use the absolute value of $\epsilon_{\text{rel}}$ to make for easier comparison across samples.

**Figure 1.**
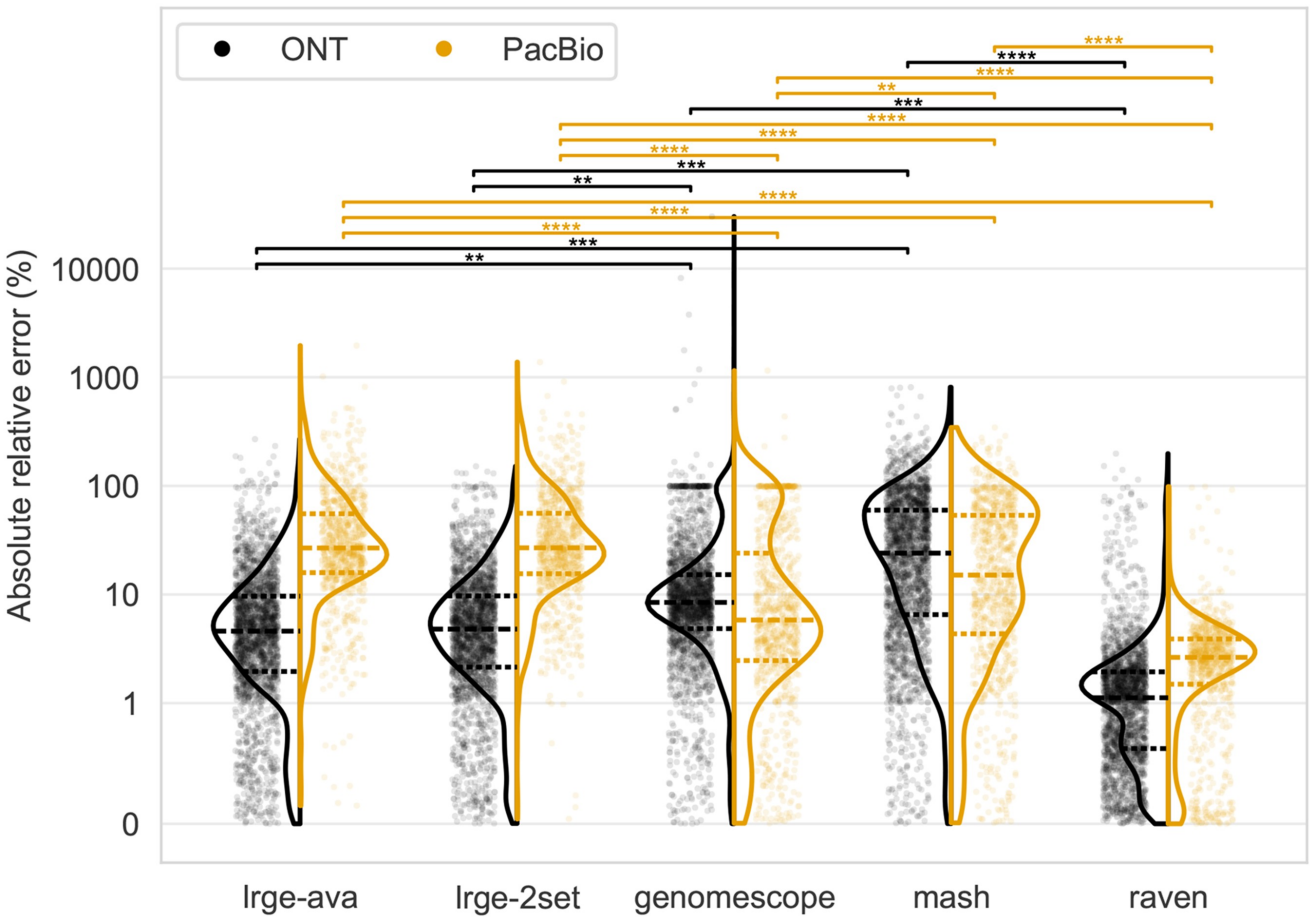
Absolute relative error (*y*-axis) for each method’s (*x*-axis) genome size estimation on ONT and PacBio data. The *y*-axis is scaled according to a symmetric logarithm, which is linear between −1 and 1 and logarithmic (base 10) thereafter. The statistical annotations are the result of a Tukey’s range test and are coloured by the sequencing platform being compared. Pairs with no annotation indicate no significant difference. The dashed lines in the violins are the quartiles.

Figure 1 shows the results of this comparison. The first observation is that the two LRGE strategies provide very similar estimates, with no significant difference. Second, LRGE and Raven perform better with ONT data than PacBio, while Mash and GenomeScope2 are the opposite. Raven provides the best genome size estimates, being statistically significantly better than all methods on PacBio data (mean: 3.5%, median: 2.6%), and GenomeScope2 and Mash on ONT data (mean: 2.9%, median: 1.1%). LRGE had significantly lower $\epsilon_{\text{rel}}$ compared to GenomeScope2 and Mash for ONT data (*ava*: mean 9.2%, median 4.6%; *2set*: mean 8.9%, median 4.8%), though the inverse was true for PacBio data. The full results can be seen in Table 1, available as supplementary data at *Bioinformatics* online.

**Table 1.** Estimates on eukaryote samples.^a^

Organism	Ploidy	Chromosomes	**True size** ^b^	Method	**Estimate** ^b^	$\epsilon_{\text{rel}}$ ^c^	**Time** ^d^	Memory (GB)
*Saccharomyces cerevisiae*	1	16	12.2 Mbp	LRGE *2set*	13.8 Mbp	13.76	0:21:56	4.7
				LRGE *ava*	14.1 Mbp	15.73	0:12:05	5.3
				Mash	55.7 Mbp	358.48	**0:08:13**	**0.9**
				GenomeScope2	15.1 Mbp	23.96	0:17:09	11.2
				Raven	13.0 Mbp	**7.14**	3:20:32	51.3
*Drosophila melanogaster*	2	7	143.7 Mbp	LRGE *2set*	144.8 Mbp	**0.77**	1:47:05	16.7
				LRGE *ava*	145.9 Mbp	1.55	1:25:55	21.3
				Mash	135.6 Mbp	−5.64	**0:07:27**	**1.8**
				GenomeScope2	150.6 Mbp	4.76	0:15:27	11.2
				Raven	147.7 Mbp	2.79	1:45:58	51.3
*Arabidopsis thaliana*	2	5	119.7 Mbp	LRGE *2set*	143.1 Mbp	19.55	12:00:15	29.4
				LRGE *ava*	143.5 Mbp	19.93	9:42:23	44.2
				Mash	147.2 Mbp	22.96	**0:16:02**	**1.8**
				GenomeScope2	152.9 Mbp	27.74	0:27:45	11.0
				Raven	123.5 Mbp	**3.16**	7:09:10	56.8
*Homo sapiens*	2	24	3117.3 Mbp	LRGE *2set*	2807.8 Mbp	−9.93	194:34:48	46.3
				LRGE *ava*	–	–	–	–
				Mash	2307.0 Mbp	−25.99	**1:24:03**	**1.8**
				GenomeScope2	2962.3 Mbp	−**4.97**	2:55:47	11.3
				Raven	2900.5 Mbp	−6.95	365:38:27	112.6

a Bold text indicates the best value for the relevant metric for each organism.

b Size values have been rounded to megabase pair with one decimal place for compactness.

c $\epsilon_{\text{rel}}$ is relative error expressed as a percentage, as defined in Equation (5).

d CPU time formatted as [h]:mm:ss.

A key observation is that LRGE performs substantially better on ONT data than on PacBio. This is likely due to lower read quality in the available PacBio datasets compared to ONT, as shown in Fig. 6, available as supplementary data at *Bioinformatics* online. Notably, many PacBio reads have quality scores of zero—likely placeholders resulting from FASTA-to-FASTQ conversion during submission to SRA/ENA. Additionally, both read length and quality significantly affect estimation accuracy, with longer and higher-quality reads leading to lower $\epsilon_{\text{rel}}$ (Fig. 7, available as supplementary data at *Bioinformatics* online). For reads of equivalent length and quality, $\epsilon_{\text{rel}}$ tends to be lower for ONT data than for PacBio. This suggests that read quality alone does not fully account for the performance gap, and we were unable to determine the underlying cause of this difference.

### 3.1 Outliers

GenomeScope2 and Raven tended to underestimate the genome size (Fig. 4, available as supplementary data at *Bioinformatics* online) on ONT data, though the median was approximately −1% for Raven. In terms of outliers for LRGE there were two common patterns. Major underestimates were found to be cases where there was dramatically disproportionate depth across the genomes. In one example, a *Pandoraea fibrosis* run (SRR9733840), the ribosomal RNA genes had up to 100 000× read depth. In another, a *Enterobacter ludwigii* run (SRR12247681) had two plasmids with 50 000× and 160 000× depth. This dramatic difference in copy number for plasmids is a known mechanism of transient antibiotic heteroresistance (Nicoloff *et al.* 2024). These dramatic depth differences lead to underestimates because, when randomly selecting reads, there is a high likelihood that mostly plasmid or gene reads will be selected, and thus the estimate will reflect the (much smaller) size of the plasmid(s) or gene(s).

The other major outlier pattern was low quality runs leading to large overestimates. This is illustrated in Fig. 5, available as supplementary data at *Bioinformatics* online where we see runs with a $\epsilon_{\text{rel}}$ over 50% with average read qualities much lower than other samples. Assumably this lower quality makes it very difficult to overlap reads, leading to fewer overlaps, and thus larger genome size estimates.

### 3.2 Calibration of estimate confidence range

As we calculate a genome size estimate for each read, we can determine a range within which the estimate is likely to fall with a given level of confidence. To achieve this, we scanned all percentile ranges of width 50%, ensuring that the overall estimate (median) was contained within the range. Figure 8, available as supplementary data at *Bioinformatics* online illustrates this process and shows that the percentile range that maximizes the proportion of samples with the true genome size within the corresponding range is 15%–65% for both LRGE strategies. We found that 92.5% (*2set*) and 87.8% (*ava*) of samples had their true genome size fall within this estimate range.

On the prokaryote dataset, this range gave an interquantile range (IQR) of 0.53 and 0.44 relative size ($\frac{\text{estimate}}{\text{true}\;\text{size}}$) for the *2set* and *ava* strategies, respectively. For example, an IQR of 0.53 means that for the *2set* strategy, the middle 50% of estimates fall within a range that is 53% of the true genome size, while for the *ava* strategy, this range is only 44%. This illustrates that the *ava* strategy provides a more precise estimate, with less variability in the middle half of the predictions compared to the *2set* strategy.

### 3.3 Runtime and memory benchmark

**Figure 2.**
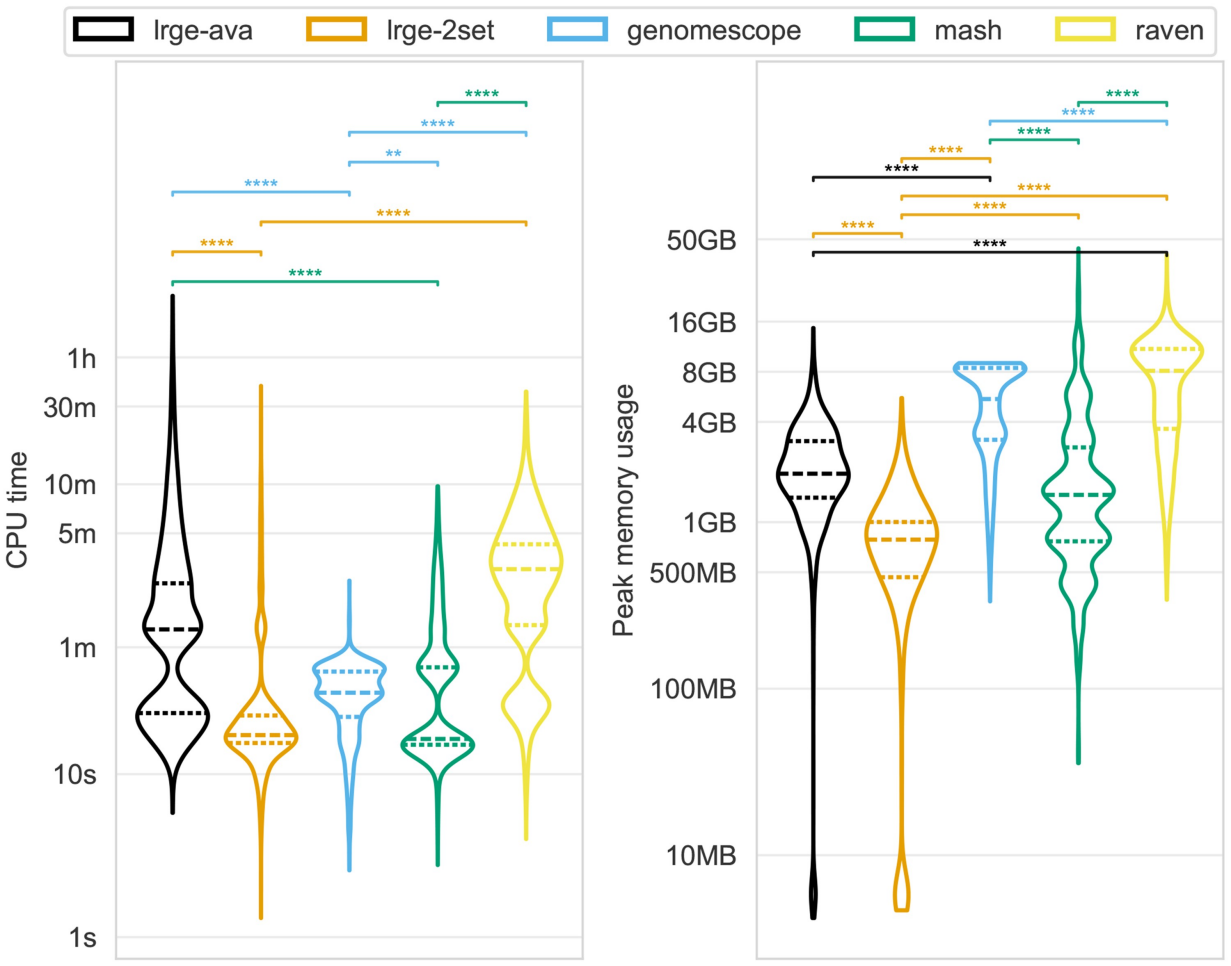
CPU time (left *y*-axis) and maximum memory usage (right *y*-axis) for each method (colours). The *y*-axis is log-scaled (base 10). The statistical annotations are the result of a Tukey’s range test and are coloured by the method with the lower mean value. The dashed lines in the violins are the quartiles.

Figure 2 shows the benchmark of CPU time and maximum memory usage for all methods. In terms of CPU time, LRGE *2set* (mean: 38.2 s, median: 17.4 s) and GenomeScope2 (mean: 31.9 s, median: 31.6 s) had the quickest runtime, with no significant difference between the two. Raven was significantly slower than all methods except LRGE *ava*. The longest recorded CPU time, 2.4 hours, was LRGE *ava*. Both LRGE strategies had large CPU time outliers, highlighted by the differences between their mean (*ava*: 194.7 s, *2set*: 38.2 s) and median (*ava*: 77.2 s, *2set*: 17.4 s). These outliers were enriched in samples with large underestimates (Fig. 9, available as supplementary data at *Bioinformatics* online). Together with the fact that samples with large overestimates tended to have very fast runtimes (Fig. 9, available as supplementary data at *Bioinformatics* online), this indicates that the runtime is proportional to the number of overlaps (see Outliers).

LRGE *2set* memory usage (mean: 776 MB, median: 749 MB) was significantly lower than all other methods, while Raven had the highest memory usage (mean: 7.2 GB, median: 7.7 GB). The highest recorded memory usage was Mash, with a maximum of 42 GB.

The full results can be seen in Table 2, available as supplementary data at *Bioinformatics* online.

### 3.4 Eukaryotes

While the primary focus and development of this work has been bacteria-focused, we investigated how LRGE generalizes to multi-chromosome and diploid organisms. We evaluated LRGE, along with the other three methods, against four model organisms: *S. cerevisiae*, *D. melanogaster*, *A. thaliana*, and *Homo sapiens* (see Eukaryote evaluation). These results are summarized in Table 1 with full details provided in Table 3, available as supplementary data at *Bioinformatics* online. They demonstrate that LRGE generalizes well to eukaryotic organisms, with all estimates closely aligning with the expected genome sizes and D. melanogaster recording the lowest $\epsilon_{\text{rel}}$. For *A. thaliana*, we used the TAIR10 genome assembly as the reference; however, this version likely underrepresents the genome’s repeat content and underestimates its true size. A recent pangenome study reports *A. thaliana* genome sizes ranging from 128 to 148 Mb, with an average of approximately 135 Mb (Lian *et al.* 2024). Based on this value, LRGE actually produces the most accurate estimate. Notably, LRGE consistently yields estimates within the range of the other three methods, highlighting its robustness.

## 4 Conclusion

LRGE provides a robust and efficient method for genome size estimation tailored to long-read sequencing technologies. By leveraging read-to-read overlap data, it circumvents the limitations of *k*-mer-based methods and performs well across diverse datasets, including bacteria and eukaryotes. LRGE outperforms existing *k*-mer-based tools on bacterial genomes in both accuracy and computational efficiency and delivers estimates comparable in accuracy to those derived from an assembly-based approach, Raven, but with significantly reduced computational resource requirements. In practice, we find that excluding short or low-quality reads prior to running LRGE can improve estimation accuracy. Despite not being specifically designed for large, repetitive eukaryotic genomes, LRGE generalizes well to these datasets, demonstrating both accuracy and robustness in its estimates. Its flexibility and scalability make LRGE a valuable addition to the bioinformatics toolkit, supporting applications in genome assembly, evolutionary studies, and sequencing depth estimation.

## Supplementary Material

btaf593_Supplementary_Data

## Data Availability

All code and metadata required to perform the analyses in this work are available on GitHub at https://github.com/mbhall88/lrge and are archived at Zenodo (Hall *et al.* 2025) or in Table 3, available as supplementary data at *Bioinformatics* online for the eukaryote samples.
