# Supplementary material for: Genome size estimation from long read overlaps
Source: Bioinformatics. 2025 Nov 6;41(11):btaf593. doi: 10.1093/bioinformatics/btaf593 (PMC12608075; doi:10.1093/bioinformatics/btaf593)
Supplement: btaf593_Supplementary_Data [file btaf593_supplementary_data.pdf]

Supplementary material for *Genome size estimation from long read overlaps*

Contents

S1 Parameter exploration **3**

S2 Dataset selection **4**

References **12**

List of Figures

S1 LRGE two-set parameter exploration. Columns are the query read set size Q, while the rows are the target read set size T. Each subplot shows the estimated genome size (y-axis) against the true genome size (x-axis), with points coloured by the strategy. ϵ_{rel} is the relative error and R^2 is the coefficient of determination. The subplot with the green border is the one that was selected as the default parameters. 5

S2 LRGE all-vs-all parameter exploration. Each subplot represents the number of reads (N) used and shows the estimated genome size (y-axis) against the true genome size (x-axis), with points coloured by the strategy. ϵ_{rel} is the relative error and R^2 is the coefficient of determination. The subplot with the green border is the one that was selected as the default parameters. 6

S3 Exploring the best-performing combination of Mash sketch size (x-axis) and minimum required copies of a k-mer to pass noise filtering (colours). The darker horizontal dashed line at $y = 0$ indicates the optimal relative error. The y-axis is scaled according to a symmetric logarithm, which is linear between -1 and 1 and logarithmic (base 10) thereafter. 7

S4 Relative error (y-axis) for each method's (x-axis) genome size estimation on ONT (black) and PacBio (orange) data. The y-axis is scaled according to a symmetric logarithm, which is linear between -1 and 1 and logarithmic (base 10) thereafter. The dashed lines in the violins are the quartiles. 8

S5 Distribution of average read quality for samples with a relative error (ϵ_{rel}) greater than 50% (black) and all other samples (orange). 9
S6 Distribution of read length and quality for all reads used by LRGE on ONT (blue) and PacBio (red) data. 9
S7 Impact of read length (left) and quality (right) on LRGE genome size estimation accuracy. Points and lines are coloured by sequencing platform. The lines indicate a linear regression fit to the points with the significance of the fit and the coefficient of determination (R^2) in the legend. Infinite estimates are those reads which have no overlaps. 10
S8 Calibrating the optimal percentile range for LRGE estimates. The x-axis represents the lower end of the range. So an x value of 10 means a percentile range of 10-60. The y-axis represents the proportion of samples for which the true genome size lies within the corresponding percentile range on the x-axis. The two colours represent the two LRGE strategies. 10
S9 Relationship between CPU time (x-axis) and relative error (y-axis) for LRGE strategies (colours). The line represents a linear regression model fit to the data. 11

S1 Parameter exploration

We explored the best combination of parameters to be used for the larger dataset in the main text, by validating on a much smaller dataset. For this, we used ONT reads from 14 different bacterial species [1]. In particular, these are R10.4.1 simplex reads basecalled with the v4.3.0 Dorado super accurate (sup) model [2].

S1.1 LRGE two-set overlaps

For the LRGE two-set (*2set*) approach, the three parameters we explore are the number of query and target reads used for the overlaps, Q and T, respectively, and the read selection strategy. For the *long* strategy, we select the $|Q|$ longest reads, Q and the next $|T|$ longest reads, T. In the *rand* strategy, we randomly select Q and T reads, ensuring these sets are disjoint, using Rasusa (v2.1.0) [3]. We then determine the overlaps using minimap2 (v2.28) [4] with the Q reads as the query and T as the target. For the *rand* strategy, we performed three replicates for each sample-Q-T combination.

The metrics we used to assess the performance of the different values of Q and T are relative error (Equation 5; ϵ_{rel}) and the coefficient of determination (R^2), with respect to the identity line, for each parameter combination. From Figure S1 we can see clearly that the *long* strategy is inferior to the *rand* strategy. $Q = 5000$ with $T = 10000$ had the best balance of R^2 and ϵ_{rel} and were therefore selected as the default.

S1.2 LRGE all-vs-all overlaps

For the LRGE all-vs-all (*ava*) approach, the two parameters we explore are the number of reads used for the overlaps, n, and the read selection strategy. For the *long* strategy, we select the n longest reads and in the *rand* strategy, we select n reads at random using Rasusa (v2.1.0) [3]. We then determine the overlaps using minimap2 (v2.28) [4] with the n reads as both the target and query. For the *rand* strategy, we performed three replicates for each sample-n combination.

From Figure S2 we can see clearly that the *long* strategy is inferior to the *rand* strategy. $n = 10000$ had the highest R^2 and closest ϵ_{rel} to zero, though $n = 25000$ was very close. We chose to use 25000 as the default because of the fact it was so close to $n = 10000$ and the fact that it performed much better on the *long* strategy.

S1.3 Mash

The parameters we explored for Mash [5] were the sketch size (`-s`) and the minimum copies of a k-mer required to pass the noise filter (`-m`). We ran the Mash subcommand `sketch` with these parameters for each of the 14 samples. Figure S3 shows the results of this parameter exploration, with a sketch size

of 100000 and minimum copies of 10 giving the tightest distribution of relative error near zero.

S2 Dataset selection

Metadata for all complete bacterial assemblies in NCBI's RefSeq (excluding MAGs) were downloaded using NCBI's `datasets` command line tool. We selected assemblies where: (i) the sequencing technology section mentions PacBio or Oxford Nanopore Technologies, (ii) the assembly release date was after 01/01/2016, (iii) CheckM contamination was below 3%, (iv) the CheckM completeness was greater than 95%, (v) the CheckM completeness percentile for the species was above 75%. Next, we gathered metadata on the sequencing runs associated with the BioSample accession for each assembly. We removed assemblies which did not actually have any long read ONT or PacBio sequencing, despite criteria (i) from above.

The long read data left after these filtering steps were downloaded using Kingfisher (v0.4; [6]). We then removed a further 1,130 sequencing runs due to low volume of sequencing reads, indicated by the readset not having sufficient reads to run LRGE *2set*, or GenomeScope2 giving an error stating it was unable to converge on an estimate (an error that typically indicates low sequencing depth). We also removed 22 runs which were actually Illumina reads, despite being labeled as PacBio or ONT on the NCBI SRA, and 16 runs for which a valid FTP address could not be found to download the data.

In the end, we were left with 3,370 long read sequencing runs.

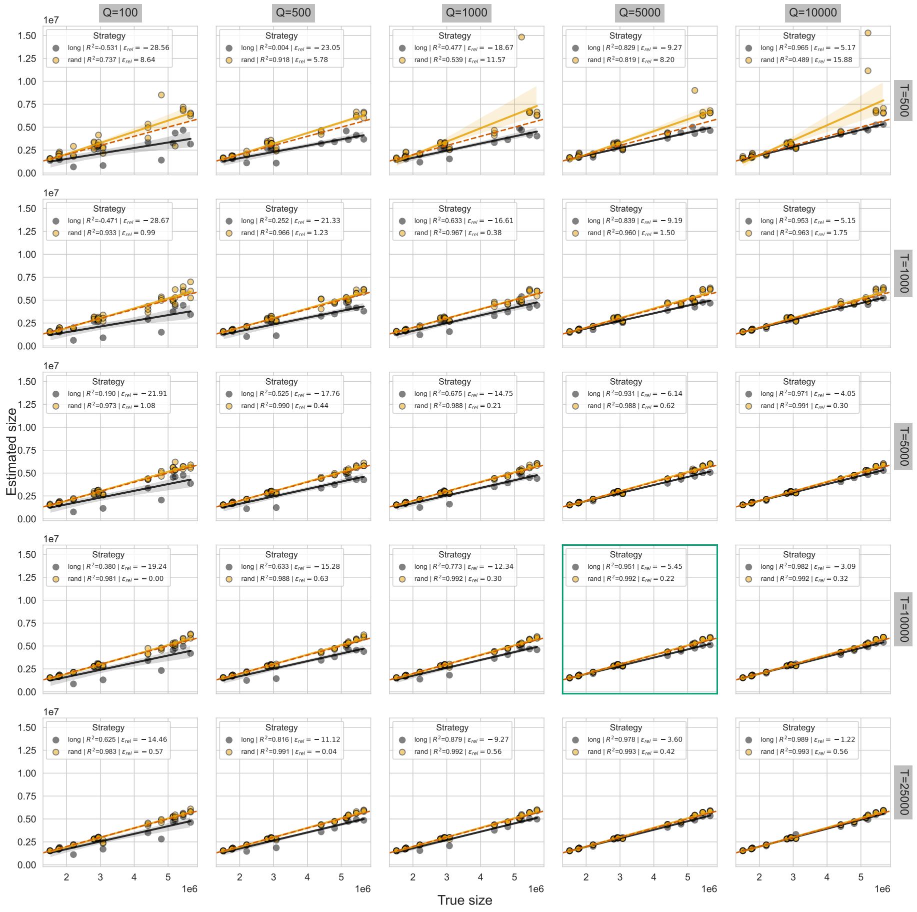

Figure S1: LRGE two-set parameter exploration. Columns are the query read set size Q, while the rows are the target read set size T. Each subplot shows the estimated genome size (y-axis) against the true genome size (x-axis), with points coloured by the strategy. ϵ_{rel} is the relative error and R^2 is the coefficient of determination. The subplot with the green border is the one that was selected as the default parameters.

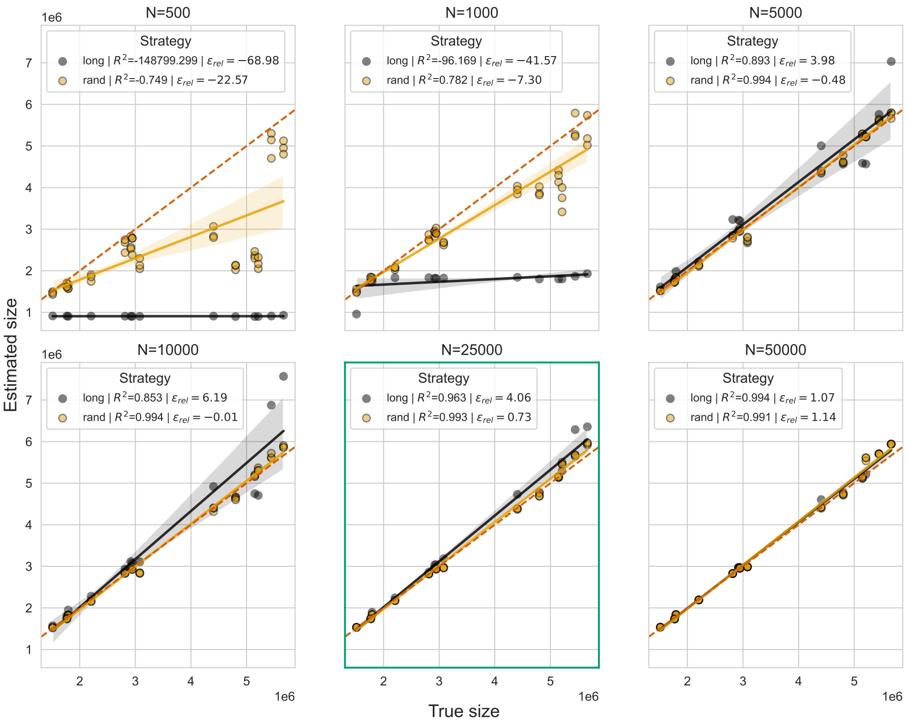

Figure S2: LRGE all-vs-all parameter exploration. Each subplot represents the number of reads (N) used and shows the estimated genome size (y-axis) against the true genome size (x-axis), with points coloured by the strategy. ϵ_{rel} is the relative error and R^2 is the coefficient of determination. The subplot with the green border is the one that was selected as the default parameters.

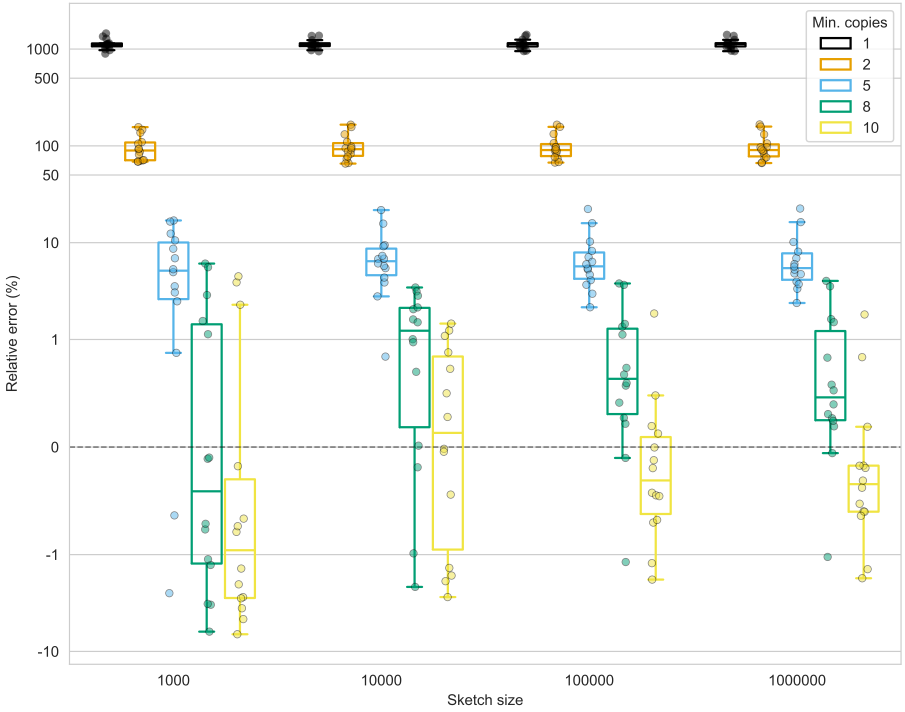

Figure S3: Exploring the best-performing combination of Mash sketch size (x-axis) and minimum required copies of a k-mer to pass noise filtering (colours). The darker horizontal dashed line at $y = 0$ indicates the optimal relative error. The y-axis is scaled according to a symmetric logarithm, which is linear between -1 and 1 and logarithmic (base 10) thereafter.

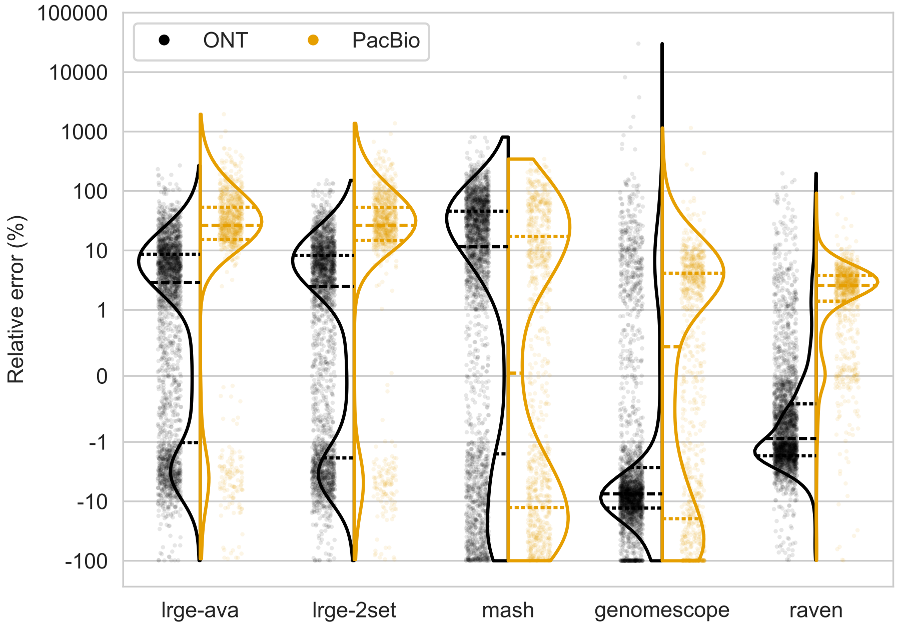

Figure S4: Relative error (y-axis) for each method's (x-axis) genome size estimation on ONT (black) and PacBio (orange) data. The y-axis is scaled according to a symmetric logarithm, which is linear between -1 and 1 and logarithmic (base 10) thereafter. The dashed lines in the violins are the quartiles.

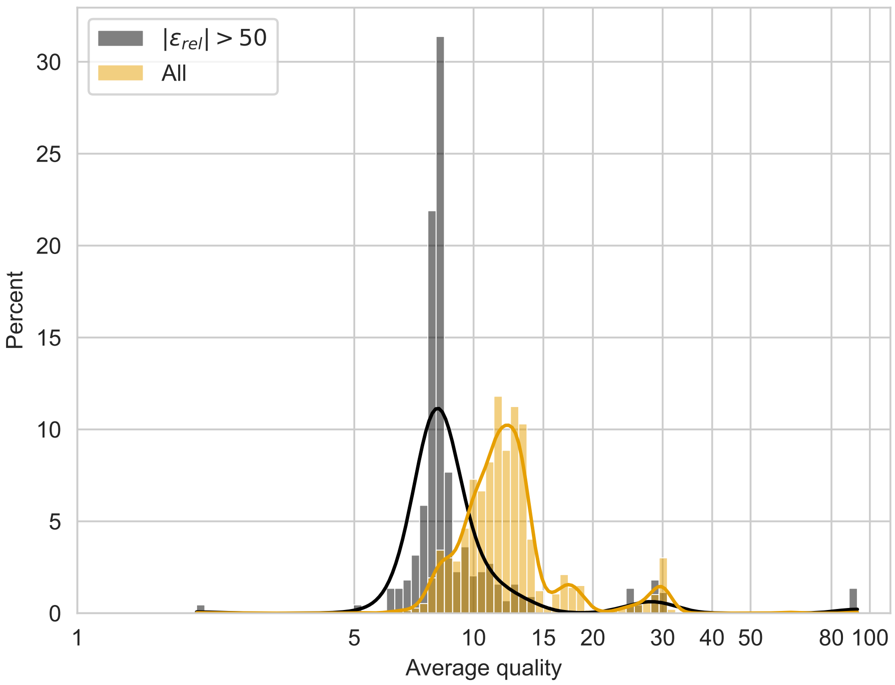

Figure S5: Distribution of average read quality for samples with a relative error (ϵ_{rel}) greater than 50% (black) and all other samples (orange).

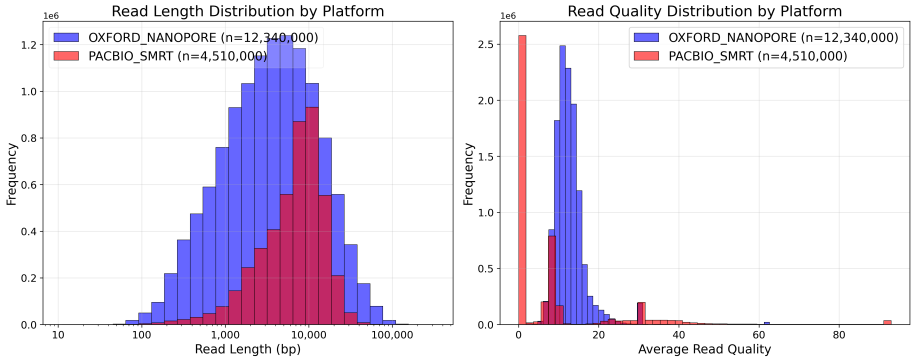

Figure S6: Distribution of read length and quality for all reads used by LRGE on ONT (blue) and PacBio (red) data.

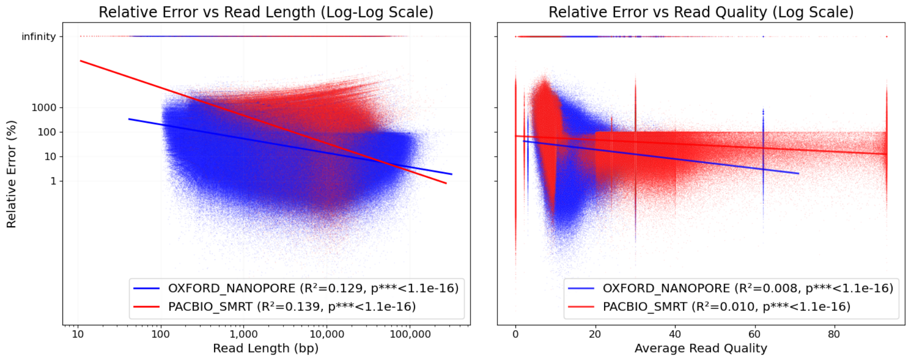

Figure S7: Impact of read length (left) and quality (right) on LRGE genome size estimation accuracy. Points and lines are coloured by sequencing platform. The lines indicate a linear regression fit to the points with the significance of the fit and the coefficient of determination (R^2) in the legend. Infinite estimates are those reads which have no overlaps.

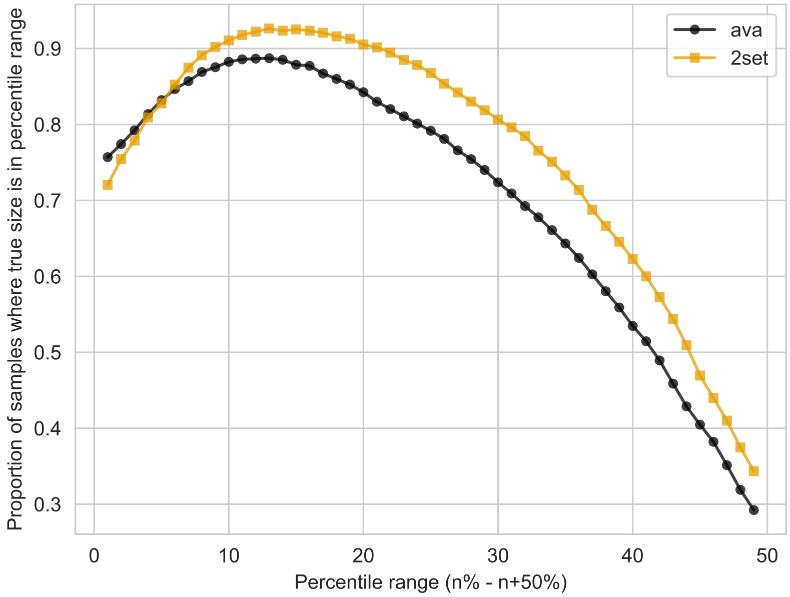

Figure S8: Calibrating the optimal percentile range for LRGE estimates. The x-axis represents the lower end of the range. So an x value of 10 means a percentile range of 10-60. The y-axis represents the proportion of samples for which the true genome size lies within the corresponding percentile range on the x-axis. The two colours represent the two LRGE strategies.

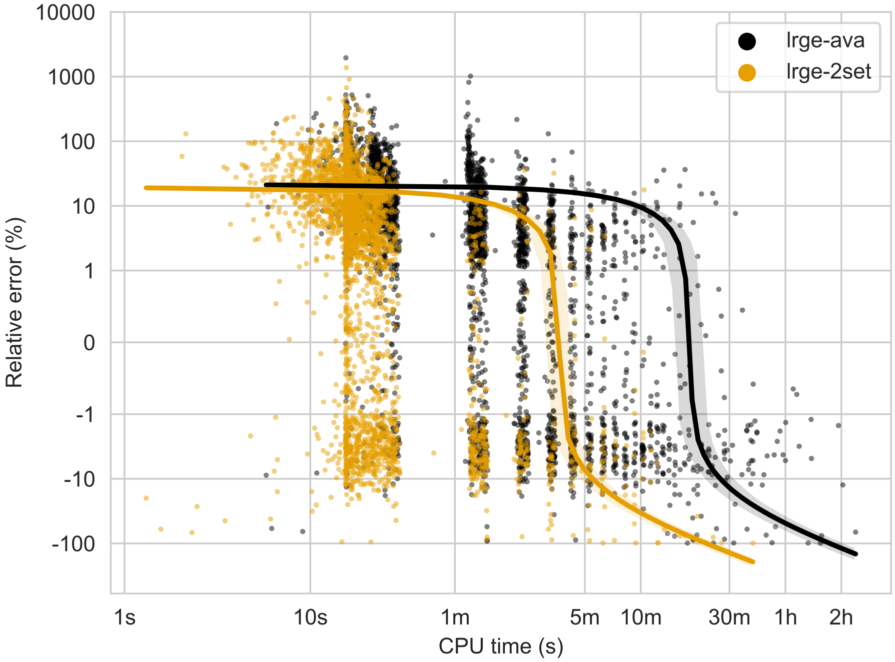

Figure S9: Relationship between CPU time (x-axis) and relative error (y-axis) for LRGE strategies (colours). The line represents a linear regression model fit to the data.

References

[1] Michael B Hall et al. "Benchmarking reveals superiority of deep learning variant callers on bacterial nanopore sequence data". In: eLife 13 (Oct. 2024). Ed. by Detlef Weigel, RP98300. ISSN: 2050-084X. DOI: 10.7554/eLife.98300. (Visited on 10/17/2024).

[2] Oxford Nanopore Technologies. Dorado: Oxford Nanopore's Basecaller. Version 0.5.0. Dec. 5, 2023. URL: https://github.com/nanoporetech/dorado.

[3] Michael B Hall. "Rasusa: Randomly subsample sequencing reads to a specified coverage". en. In: Journal of Open Source Software 7.69 (Jan. 2022), p. 3941. ISSN: 2475-9066. DOI: 10.21105/joss.03941. (Visited on 01/03/2024).

[4] Heng Li. "Minimap2: pairwise alignment for nucleotide sequences". In: Bioinformatics 34.18 (2018), pp. 3094–3100. ISSN: 1367-4803. DOI: 10.1093/bioinformatics/bty191.

[5] Brian D. Ondov et al. "Mash: fast genome and metagenome distance estimation using MinHash". In: Genome Biology 17.1 (June 2016), p. 132. ISSN: 1474-760X. DOI: 10.1186/s13059-016-0997-x. (Visited on 01/25/2024).

[6] Ben J. Woodcroft et al. Kingfisher: A utility for procurement of public sequencing data. Jan. 2024. DOI: 10.5281/zenodo.10525086.
